# Biaxial Flexural Strength of High-Viscosity Glass-Ionomer Cements Heat-Cured with an LED Lamp during Setting

**DOI:** 10.1155/2013/838460

**Published:** 2013-06-12

**Authors:** Gustavo Fabián Molina, Ricardo Juan Cabral, Ignacio Mazzola, Laura Brain Lascano, Jo E. Frencken

**Affiliations:** ^1^Department of Dental Materials, Dental Faculty, National University of Córdoba, Maya de la Torre s/n, 5000 Córdoba, Argentina; ^2^Department of Global Oral Health, College of Dental Sciences, Radboud University Nijmegen Medical Centre, P.O. Box 9101, 6500 HB Nijmegen, The Netherlands

## Abstract

Adding heat to glass ionomers during setting might improve mechanical properties. The aim was to compare the biaxial flexural strength (BFS) between and within four glass ionomers, by time of exposure to a high-intensity LED light-curing unit. *Materials and methods*. Samples of Fuji 9 Gold Label, Ketac Molar Easymix, ChemFil Rock, and the EQUIA system were divided into three treatment groups (*n* = 30): without heating (Group 1), heated with LED lamp of 1400 mW/cm^2^ for 30 s while setting (Group 2), and heated with LED lamp of 1400 mW/cm^2^ for 60 s while setting (Group 3). Samples were stored for 48 hours in distilled water at 37°C until tested. BFS was tested, using a universal testing machine at a crosshead speed of 1 mm/min. Data were analyzed, using ANOVA test with the Bonferroni correction (*α* = 0.05). Heating the glass-ionomer cements with an LED curing light of 1400 mW/cm^2^ during setting for 30 s increased the BFS value of all GICs. No statistically significant difference in mean BFS scores was found between the EQUIA system and ChemFil Rock at 30 s and 60 s. The mean BFS value was statistically significantly higher for the EQUIA system and ChemFil Rock than for Fuji 9 Gold Label and Ketac Molar Easymix at all exposure times.

## 1. Introduction

In general, the longevity of glass-ionomer cement restorations, produced using rotary instruments, are considered inferior to resin-based composite and dental amalgam restorations. However, the longevity of high-viscosity glass-ionomer cements used with the atraumatic restorative treatment (ART) protocol in permanent teeth was equal to, or greater than, that of equivalent amalgam restorations for up to 6.3 years. There was also no difference in longevity between the two types of restorations in primary teeth, assessed according to the same assessment criteria [1, 2]. A similar finding has been reported regarding the longevity of ART glass-ionomer restorations and resin-composite restorations in primary teeth assessed according to the same assessment criteria [3]. However, the number of trials, upon which the latter conclusion is based, was small. 

Glass-ionomer cements have certain features that are superior to those of resin-based materials and dental amalgam [4]. These include the following: chemical adhesion to mineralized dental tissues; biological sealing of the cavity interface (including inhibition of bacterial compounds and ability to remineralize dental tissues) [5]; and easy use in a variety of clinical settings [4]. The major weakness of glass-ionomer cements is their low fracture toughness. This feature is likely to improve as the material maturates [6, 7]. Incomplete chemical reactions and sensitivity to water during the first stage of the setting reaction of glass-ionomer cements lead to softening and cracking of the cement surface and subsequently to reduction of its wear resistance and fracture toughness [8]. 

It was thought that one way in which a solution to these adverse conditions could be achieved was to shorten the vulnerable initial stage in the setting reaction. This idea was tested to materialize with the introduction of fast-setting glass-ionomer restorative cements, but the studies did not always demonstrate higher physical-mechanical values than those of their regular set counterparts [9]. Another method that could reduce the vulnerability stage employed heat application during setting. It increased compressive strength [10, 11], decreased microleakage, and increased wall adaptation to enamel [12]. Reasons for these changes are still unknown. But it has been postulated that it might be due to changes in molecular kinetic energy that, subsequently, may lead to a rearrangement of the molecules in the material which facilitate a better adhesion of the material to tooth tissues or achieving a more stable zone of ionic exchange [12].

Heat is generated as a byproduct of LED light curing of photosensitive dental materials with a high-intensity photopolymerization device [13]. Apparently not all LED curing lights seemed to emit sufficient heat. Therefore, a special “heat-curing” LED light-curing unit has been marketed. Its output temperature reaches 60° Celsius in less than 1 minute [14]. However, this temperature might be too high for use in the oral cavity. In order to investigate the influence of heat on the mechanical strength of glass-ionomer cements, the output temperature of heat-emitting curing lights needs to be investigated. In the present study, biaxial flexural strength was chosen to represent the common mechanical strength tests, as it was found to be the most reliable and the best mechanical property test, for determining the mechanical strength of glass-ionomer cements [15, 16]. 

The aims of the present study, the first of its kind, were to compare the following: (1) the output temperatures of three LED light-curing units, (2) the biaxial flexural strength of four high-viscosity glass-ionomer cements treated with the selected heat source by curing time, and (3) the effect of light-curing time on the biaxial flexural strength of the tested glass-ionomer cements.

## 2. Materials and Methods

### 2.1. Testing Curing Light Output Temperature

The tests were carried out by one senior investigator (Gustavo F. Molina) assisted by two colleagues from the same department (Ignacio Mazzola and Laura Brian Lascano) in the laboratory of the Dental Materials Department of the Dental Faculty, University of Córdoba, Argentina.

Three LED light-curing units were selected from the ones available at the dental Faculty. These were as follows: GSK-Densell LED555 (Dental Medrano S.A., Buenos Aires, Argentina) with a light intensity of 800 mW/cm^2^; ECCO-Light (SD Dental, Córdoba, Argentina) with a light intensity of 1400 mW/cm^2^; and GCP CarboLED CL-01 (GCP Dental, Vianen, The Netherlands) with a light intensity of 1400 mW/cm^2^. The latter device was specially designed to generate heat. 

The output temperature was measured every ten seconds during a one-Minute period, using a thermometer filled with red ethanol (LED Lamp Test kit, GCP Dental, Elmshorn, Germany) and placed at the tip of each curing light. On the basis of the results of this test, the ECCO-Light lamp was selected for testing the biaxial flexural strength of the four glass-ionomer cements (Table 2).

### 2.2. Testing Biaxial Flexural Strength (BFS)

The four glass-ionomer cements tested were the following: Fuji 9 Gold Label (GC Europe, Leuven, Belgium); Ketac Molar Easymix (3 M ESPE, Seefeld, Germany); ChemFil Rock (Dentsply-DeTrey, Konstanz, Germany); and the EQUIA system (GC, Tokyo, Japan). Particulars of these cements are listed in Table 1. Thirty specimens, each 2.1  (±0.5) mm thick and 13  (±0.5) mm in diameter, were prepared for each of the four glass-ionomer cements. 

Three treatment protocol groups were established: (1) auto-curing (no light-curing device) (SC) as the control; (2) light curing with ECCO-Light lamp during 30 s, starting immediately after the disc was filled (HC30); and (3) light curing with ECCO-Light lamp during 60 s, starting immediately after the disc was filled (HC60). 

Disc-shaped samples were prepared, using polytetrafluoroethylene ring moulds placed on a polished glass slab covered with an acetate strip. To be able to fill one mould with the hand-mixed glass-ionomer cements (Fuji 9 Gold Label and Ketac Molar Easymix), two operators simultaneously mixed three portions (1 ∶ 1) powder and liquid each, according to the manufacturers' instructions. After mixing, the cements were inserted into the middle of the mould with a spatula and covered with a second acetate strip. Then a 1 kg glass slab was placed on top of the glass-ionomer cement, to spread the material evenly throughout the ring. 

For treatment protocol Groups 2 and 3, the glass slab was removed after 5 s, but the acetate strip was left on top. The ECCO-Light lamp was placed on top of the glass-ionomer cements, making contact with the acetate strip, and the material was light-cured either for 30 s (Group 2) or 60 s (Group 3). After heat-curing, the glass slab was again placed over the mould. For the encapsulated glass-ionomer cements (ChemFil Rock and the EQUIA system), two capsules were needed for filling the mould. Each capsule was tumbled for 5 s before activation, to aerate the powder inside the capsule. Capsules were activated according to the manufacturers' instructions, by pushing the extension into the capsule and then squeezing the extruder once to break the seal. After activation, the first capsule was inserted into a mixing device (Ventura Mix III, Madespa S.A., Spain). A second mixing device of the same brand was used in activating the second capsule. The ChemFil Rock capsules were mixed for 15 s and the EQUIA system capsules for 10 s. The first capsule of each material was extruded into the centre of the mould, using an applicator extruder (Dentsply-DeTrey, Konstanz, Germany). The mix of the second capsule followed immediately. From this point onwards, the procedure was identical to the one described for heat-curing of treatment protocol Groups 2 and 3.

Each batch, containing ten disc moulds and covered underneath and above by a glass slab, was secured with clamps and immersed in a water bath at 37 ± 1°C for one hour. Specimens were then removed from their moulds and stored in 50 mL distilled water at 37 ± 1°C for an additional 47 hours. Before storage, all specimens of the EQUIA system were coated with a nanofilled resin (G-Coat, GC, Tokyo, Japan) and light-cured for 10 s using the same ECCO-Light  lamp. 

Specimens were placed on a 10 mm diameter knife-edge circular support covered with thin rubber. The BFS tests were performed with a universal testing machine (Digimess MX5000) at a crosshead speed of 1 mm/min, using a 4 mm diameter ball indenter, loading the specimens centrally. The BFS value was calculated according to the following equation [17]: BFS = *P*/*h* ^2^((1 + *v*)(0.485ln⁡(*a*/*h*) + 0.52) + 0.48), where *P* is the load at fracture, *a* is the radius of the support (5 mm), *v* is the Poisson ratio (0.3 for glass-ionomer restorative cements) [18], and *h* represents the thickness of the specimen, obtained by the mean thickness value of the two remaining fragments of each disc that is fractured when loaded.

### 2.3. Statistical Analyses

Data were analysed by a statistician using SAS 9.0 (SAS Corporation Inc., Cary, USA). The chi-square test was used for comparing the output temperatures of the light-curing devices (independent variable), with time of exposure (0 s to 60 s) being the dependent variable. ANOVA test and the Bonferroni correction were used in testing the biaxial flexural strength (BFS) (dependent variable) of the four glass-ionomer cements according to time of exposure (independent variables). A statistically significant difference was set at *α* = 0.05.

## 3. Results

### 3.1. Output Temperatures

Table 2 shows the mean output temperatures of the three LED curing units by time exposure. The ANOVA test showed an effect of exposure time (*P* < 0.0001) and curing light (*P* < 0.0001) on the output temperature. The highest mean output temperature at 30 s and 60 s was obtained for the GCP lamp, while the GSK lamp showed the lowest mean output temperatures at these time points. The output temperature for the GCP lamp increased statistically significantly between 10 s and 30 s and between 30 s and 60 s (*P* = 0.05; Bonferroni). At all exposure times, the output temperature of the GCP lamp was statistically significantly higher than that of the ECCO-Light lamp (*P* = 0.05; Bonferroni), whilst the output temperature of the ECCO-Light lamp was statistically significantly higher than that of the GSK lamp at time points 10 s up to 40 s (*P* = 0.05; Bonferroni). The output temperature of the GCP lamp at 10 s was approximately equal to that of the ECCO-Light lamp at 30 s. 

### 3.2. Biaxial Flexural Strength between Glass Ionomers by Exposure Time

The mean BFS scores and standard error for the four glass-ionomer cements, light-cured by the ECCO-Light lamp (1400 mW/cm^2^), are presented according to exposure time in Table 3. The ANOVA test showed an effect of the glass-ionomer cements (*P* < 0.001) and of curing time (*P* < 0.001) on the mean BFS scores. The autocured EQUIA system had statistically significantly higher mean BFS scores than the other three glass-ionomer cements, while Ketac Molar Easymix had the lowest mean BFS scores (*P* = 0.05; Bonferroni). There was no statistically significant difference in mean BFS scores between the EQUIA system and ChemFil Rock at 30 s and 60 s (*P* > 0.05). The mean BFS scores for the hand-mixed glass-ionomer cements were statistically significantly lower than those for the encapsulated glass-ionomer cements when autocured and light-cured for 30 s and 60 s (*P* = 0.05; Bonferroni).

### 3.3. Biaxial Flexural Strength within Glass Ionomers by Exposure Time

A time effect was observed for the mean BFS scores of all glass-ionomer cements tested (*P* < 0.0001). The mean BFS scores of all glass-ionomer cements at 30 s were higher than those of the autocured glass-ionomer cements of the same brand (*P* = 0.05; Bonferroni). Only the EQUIA system and Ketac Molar Easymix had higher mean BFS scores at 60 s than at 30 s (*P* = 0.05; Bonferroni) (Table 3). 

## 4. Discussion

### 4.1. Methodological Aspects

In the present study, selection of the heating source and determination of the heat exposure time required a critical balance between what is considered a clinically feasible and an acceptable temperature rise within one minute of activation. Heat-curing the samples of four conventional glass-ionomer cements, between two metals at 70°C for 5 min, at 15 min, over 1 and 24 hours and 28 days, resulted in a significant improvement in the compressive strength [10]. This experiment provided evidence that heating glass-ionomer cements increased the mechanical strength. It also showed that a different heat source, other than metal plates, that would be clinically acceptable and would operate at a lower temperature and over a shorter exposure time was needed. These conditions were found in the LED curing light with 1400 mW/cm^2^. The selection of 30 s and 60 s was guided by the following studies. Heating glass-ionomer restorative cements with an LED light-curing unit of 1200 mW/cm^2^ during 40 s improved marginal adaptation to enamel [12]. The same exposure time was used to heat cure glass-ionomers with a halogen light, improving the hardness of the upper and lower surfaces of the samples [19]. As care should be taken when applying heat sources in the oral cavity, the decision was made to lower the time exposure from 40 s to 30 s, while using a light-curing device with an increased intensity.

Although the output temperature for the GCP lamp at 10 s hardly differed from that for the ECCO-Light lamp at 30 s, the ECCO-Light lamp was selected for the BFS experiments in the present study. Reasons included the following: the recommendation stated in the Instructions for Use of the GCP lamp to heat-cure glass carbomer, a cement that is chemically related to high-viscosity glass-ionomer cements, for 60 s, that the output temperature of the GCP lamp at 60 s and even at 30 s was above 50°C. The latter was considered to be too high as it might cause discomfort to patients. Another consideration was that such a high temperature would create adverse changes, due to dehydration, in the microstructure of glass-ionomer cements [20]. 

A number of standard tests such as those covering compressive, diametral tensile, and flexural strengths have been used for testing mechanical properties of glass-ionomer cements. In the present study, the biaxial flexural strength test was selected owing to its relatively simple and accurate procedure for preparing the specimens. That reduces the operator-induced variability and improves the standard for assessing mechanical properties of glass-ionomer cements [15, 16]. Besides that, the BFS test has the advantage that it uses a knife-edge circular support covered with rubber, providing a platform that allows even distribution of the load in the sample, and bends to its maximum capacity without crack formation, expressing the mechanical integrity of the material until it fractures. Moreover, loading the sample with the ball-shaped indenter is suggested to be an appropriate procedure for managing crack formation associated with the brittleness of these ceramic cements in mechanical tests, in a similar fashion to the “ball on disc” protocol advocated by Darvell [21]. 

Preparation of the samples presented a challenge because the glass-ionomer cements set so fast that there was hardly sufficient time for filling the moulds. That two capsules were needed to fill the mould completely aggravated the filling process. Furthermore, condensation of the glass-ionomer cements with the glass slab took place for a few seconds only, as it had to be interrupted for either 30 or 60 seconds for the heat application process in Groups 2 and 3. This fast sequence of steps in the preparation of the samples might have created voids in the materials. Owing to these two factors, a considerable number of samples had to be discarded. 

### 4.2. Study Findings

Heating all four glass-ionomer cements with the 1400 mW/cm^2^ LED curing light for 30 s increased the biaxial flexural strength. The mean BFS values were significantly higher for the encapsulated than for the hand-mixed glass-ionomers. The EQUIA system and Ketac Molar Easymix showed significantly higher mean BFS values at 60 s than at 30 s. As very few studies have investigated the effect of heating glass ionomers with high-intensity curing lights and, in particular, using the biaxial flexural strength test, it is difficult to compare the findings of this study with those of others.

The same protocol for testing the BFS for the same type of glass ionomers was strictly followed, but the mean BFS values obtained in the present study for Chemfil Rock were somewhat lower than those reported by Fleming et al. [16]. Difficulties experienced in preparing the samples of Chemfil Rock may account for this difference. Achieving an even distribution of the content of the two capsules used in one mould turned out to be very difficult, as the setting of the first material occurred so fast that the glass ionomer was already reasonably hard at the time when the content of the second capsule was extruded into the mould. 

The effect of heat application, through use of an LED 1200 mW/cm^2^ curing light in three 20-second periods during setting (60 seconds in total), on the flexural strength, modulus of elasticity, and micromechanical behavior of glass carbomer cement, a restorative material chemically associated with conventional glass-ionomer cements, did not show an improvement in mechanical properties [22]. This finding is different from that of the present study and might be due to the chemical composition of the glass carbomer and conventional high-viscosity glass-ionomer cement tested [22]. 

 Improving the physical-mechanical properties of restorative glass-ionomer cements has been a great challenge for researchers. Introduction of new glass fillers, nanotechnology, modified liquid formulas, and other innovations has been investigated in order to reach the material's maximum potential as a valid alternative of dental amalgam or even resin-based composites [23]. This challenge is not unrealistic as the treaty of the UNEP (United Nations Environmental Programme) on amalgam pollution includes a statement related to increasing caries prevention and related to increasing research on alternatives to amalgam [24]. Heat curing glass-ionomers through use of a high-intensity curing light in order to accelerate the setting reaction, and subsequently the maturation of the material, might contribute to enhancement of the mechanical performance, particularly for the newer encapsulated glass-ionomers EQUIA system and Chemfil Rock. Whether this increase in mechanical strength is sufficient to fulfill the requirements for substitution of dental amalgam remains to be seen. Clinical acceptance of the heating procedure tested, using the EQUIA system and ChemFil Rock in the conventional restorative and ART protocols, should be evaluated. 

## 5. Conclusions

Heating the restorative glass-ionomer cements with an LED curing light of 1400 mW/cm^2^ during setting for 30 s increased the BFS value of all materials tested. The mean BFS value was significantly higher for the EQUIA system and ChemFil Rock encapsulated glass ionomers than for the Fuji 9 Gold Label and Ketac Molar Easymix hand-mixed glass ionomers at all exposure times.

## Figures and Tables

**Table 1:** Product name, manufacturer, composition, batch number, expiration date, and shade of the glass ionomers tested.

Product name	Manufacturer	Components	Batch no.	Expiry date	Shade
Fuji 9 Gold Label	GC Europe (Leuven, Belgium)	Powder: fluoroaluminosilicate glass, polyacrylic acid powder Liquid: polyacrylic acid, polybasic carboxylic acid	N219047	2013/11	A3
Ketac Molar Easymix	3M ESPE (Seefeld, Germany)	Powder: Al-Ca-La fluorosilicate glass, 5% copolymer acid (acrylic and maleic acids) Liquid: polyalkenoic acid, tartaric acid, water	406641	2016/06	A3
ChemFil Rock	Dentsply DeTrey GmbH (Konstanz, Germany)	Zinc-modified fluoroaluminosilicate glass polyacrylic and itaconic acids	103000542	2014/02	A3
EQUIA system (Fuji GP Extra + G-Coat)	GC Asia (Tokyo, Japan)	Fuji 9 GP Extra: water, fluoroaluminosilicate glass, polybasic carboxylic acid, polyacrylic acid G-Coat: methyl methacrylate, colloidal silica, camphorquinone, urethane methacrylate, phosphoric ester monomer	0903039	2012/11	A3

**Table 2:** Mean and standard error (SE) of the output temperature in °C for the three curing lights, by time.

Lamp	Time (sec)											
	10		20		30		40		50		60	
	Mean	SE	Mean	SE	Mean	SE	Mean	SE	Mean	SE	Mean	SE
ECCO	35.0^a^	0.6	39.8^d^	0.9	41.0^g^	1.0	42.8^j^	1.8	42.5^m^	2.6	42.0^o^	2.9
GCP	41.3^b^	1.1	48.0^e^	0.8	53.3^h^	0.6	54.0^k^	1.2	56.0^n^	1.6	57.3^p^	2.4
GSK	32.0^c^	0.0	34.5^f^	0.3	34.5^i^	0.7	36.3^l^	0.5	37.0^m^	0.6	35.0^o^	0.4

The number of samples is 12 per exposure time. Different superscript letters show significant statistical differences (the Bonferroni test) between the materials.

**Table 3:** The mean biaxial flexural strength (BFS) values expressed in MPa and standard error (SE) of the glass ionomers tested, by test group.

Glass ionomer	Group 1 (SC)		Group 2 (HC30)		Group 3 (HC60)	
	Mean	SE	Mean	SE	Mean	SE
Chemfil Rock	61.4^d^	1.1	73.1^e^	1.2	75.5^e^	1.3
EQUIA system	67.9^d^	1.1	73.1^e^	1.3	78.1^f^	1.1
Fuji 9 Gold Label	43.4^a^	0.6	52.1^b^	1.2	54.5^b^	1.1
Ketac Molar Easymix	39.6^a^	0.6	51.7^b^	1.1	55.2^c^	1.1

SC: autocure; HC30: light cured for 30 seconds; and HC60: light cured for 60 seconds.

The number of samples is 30 per glass-ionomer group. Different superscript letters show significant statistical differences (the Bonferroni test) within the materials.
